# Mortality in Germany during the COVID-19 Pandemic

**DOI:** 10.3390/ijerph20206942

**Published:** 2023-10-19

**Authors:** Alois Pichler, Dana Uhlig

**Affiliations:** Faculty of Mathematics, University of Technology Chemnitz, 09111 Chemnitz, Germany; alois.pichler@math.tu-chemnitz.de

**Keywords:** excess mortality, parametric mortality models, improving mortality rates, COVID-19

## Abstract

Is there sufficient scientific evidence for excess mortality caused by COVID-19? The German population, similar to the population of many other countries, is subject to fluctuations caused by multiple factors, including migration and aging. COVID-19 is one additional factor, superposing natural or seasonal mortality fluctuations. To give scientific evidence for excess mortality caused by COVID-19, it is essential to employ appropriate statistical tools. This study develops a score indicating excess mortality and studies its evolution over time. Applied to data provided by governmental authorities, the indicator discloses, without relating to causes of death explicitly, excess mortality at the end of 2020, in 2021, and in 2022. In addition, the indicator confirms that COVID-19 particularly impacted the elderly segment of the population.

## 1. Introduction

This paper strives for a detailed analysis of excess mortality in Germany during the Corona pandemic. Building on historical population statistics as well as official death counts, the analysis employs a dedicated indicator to identify and substantiate excess mortality.

Important institutions such as the German Federal Statistical Office compare the number of deceased during COVID-19 seasons with the observed mean or median number of deaths in previous years (cf. [1]). From these numbers, it seems evident that there were more deaths during the COVID-19 seasons than in preceding years. However, comparing the number of deceased in different years is problematic and questionable for several reasons. The age profile of the population, for example, varies over time. Strong influenza waves (cf. [2,3]) influence the comparison as well. The flu epidemic in the first weeks of 2018, e.g., the most severe ever in Germany, was accompanied by an extreme increase in deaths. Comparing pure death counts with the average counts of previous years is therefore not sufficient to substantiate excess mortality.

Parametric mortality models generally enjoy a high precision (cf. Zeng et al. [4] for the four- and five-parameter lifetime distribution; Missov et al. [5] and Cohen et al. [6] for Gompertz’s force of mortality involving the mode age at death). Precise and accurate estimates for small portions of the population are more challenging, for example, for elderly people, who seem to be affected and concerned most by COVID-19. Before the COVID-19 crisis, Gavrilov and Gavrilova [7] investigated human death rates of advanced ages. They do not find deviations from Gompertz’s law, not even for very old people. In fact, their abstract states:

*Study of several single-year extinct birth cohorts shows that mortality trajectory at advanced ages follows Gompertz’s law up to the ages 102–105 years without a noticeable deceleration.*

Our model incorporates the life expectancy, which is increasing year by year, in addition, since the COVID-19 pandemic occurred after several decades of continually increasing life expectancy.

Numerous studies analyzing similar or related questions are based on regression models (cf. [8,9,10] or [11]) or comparisons of death counts before and during the pandemic (cf. [12,13]). Contrary to many other investigations, this study builds on an indicator, which is suitable to disclose excess mortality. The main idea of the indicator is to adequately relate observed mortality rates with estimated ones. For this, it is essential to estimate mortality rates without COVID-19 from historical data, and to compare them with observed mortality rates during seasons with COVID-19. In fact, we will detail below that the indicator discloses excess mortality during the COVID-19 pandemic, especially among the elderly.

Many recent studies incorporate clinical data (like [9,14,15] or [16]), but these are not publicly available for the whole of Germany. The basis of the comparison is an extrapolated German life table, published by official authorities, as well as a supplementary life table, which is based on data from 1990 to 2017.

COVID-19 mortality has been studied since the first appearance of the virus. For comparable literature on national COVID-19 mortality rates, we may refer to El Fatinim et al. [17], Kowall et al. [12], and Cairns et al. [18]. Our results complement this existing stream of literature for Germany.

We estimate the baseline weekly mortality in 2020–2022 using a parametric model, which includes the life-expectancy improvements in recent years. A dedicated indicator substantiates excess mortality during COVID-19 seasons, particularly for elderly persons.

## 2. Materials and Methods

### 2.1. Available Data

The human mortality database provides access to documented deaths for different populations, mainly of Western countries (https://mortality.org/ (accessed on 16 October 2023)). The webpage https://mpidr.shinyapps.io/stmortality/ (accessed on 16 October 2023) provides an impressive visualization tool as well (cf. Németh et al. [19]); or in Our World in Data (https://ourworldindata.org/grapher/excess-mortality-raw-death-count (accessed on 16 October 2023)).

The German Federal Office (cf. German Federal Statistical Office: Statistisches Bundesamt (Destatis) [20] and the German Federal Statistical Office: Population data by Statistisches Bundesamt (Destatis) [21,22]) report even weekly data of deaths, adjusted life tables, and population updates. Our analysis combines both sources. These sources report more deaths in the COVID years 2020, 2021, and 2022, which gives evidence for excess mortality; however, this observation does not prove excess mortality, specifically not for specific ages.

Compared to other populations, the German population is specific and unique for historical and political reasons. Most individuals aged 35 or older were born in either the German Democratic Republic or the Federal Republic of Germany: two different countries, with differing mortality. For this reason, we confine our data to the fall of the Iron Curtain.

### 2.2. Mortality Model

Several mathematical models have been investigated in the academic (and particularly the actuarial) literature to capture the peculiarities and specific features of mortality rates, which vary with age, sex, progress in medicine, and presumably regions.

The literature distinguishes parametric and non-parametric mortality models. Non-parametric models do not presume a specific shape of the distribution, and therefore are more general and consequently require more data than parametric models to achieve comparable precision. To estimate mortality with high precision, specifically for older ages, we employ a parametric model and provide evidence for the model selection. Our model accounts for the constant improvement of mortality over time, which has been empirically observed during recent decades.

#### 2.2.1. Parametric Mortality Model

Traditional life tables model the force of mortality (also known as hazard rate, or hazard function in reliability theory), that is, the instantaneous rate of mortality at a certain age on an annual basis. The force of mortality of the well-established Makeham (or Gompertz–Makeham) model can be stated as

(1) $$\begin{array}{cc} \mu_{\vartheta }\left(x\right) & =\alpha +\beta \;e^{\beta (x-M)}, \end{array}$$

where *x* is the individual’s age in years, from age x=0 (infant) up to the age x=80 and further. The term α, termed *Makeham term* (cf. [5]), models age-independent causes of death such as accidental death or serious illness. The parameter β is the rate of how mortality increases year by year of life. The parameter *M* has the natural interpretation of the mode age of death of the population, that is, the age with the highest probability to die. The vector comprising all parameters in (1) is ϑ:=(α,β,M). Based on the force of mortality, the age-specific annual mortality rates are $q_{\vartheta ;x}:=1-\exp\left(-\int_{x}^{x+1}\mu_{\vartheta }\left(s\right)\;ds\right)$, as detailed in (A1) in the Appendix A (cf. also Richards [23]).

#### 2.2.2. Decreasing Mortality, Year by Year

**Figure 1:**
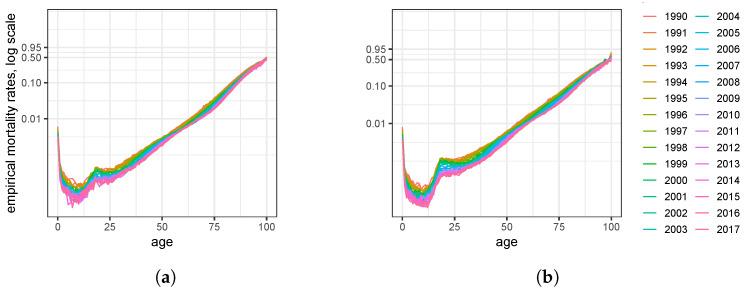
German empirical mortality rates ($q_{x}$ for all ages on logarithmic scale) providing evidence for parametric model (2): (**a**) women; (**b**) men.

COVID-19 appears at the end of a period with constantly increasing life expectancy. Improving mortality is critical for our purpose to estimate the number of deaths as precisely as possible. For this, the mortality trend thus must not be neglected. Figure 1 below depicts empirical mortality rates *q_x_* for ages x=0 up to x=100, and different calendar years. The figure apparently exhibits linear mortality improvements on the logarithmic scale from age 25 on.

Cohen et al. [6], Osmond [24] and [Sections 2.3 and 7.5.4] in Keyfitz and Caswell [25] investigate the evolution of a population’s mortality over time, as well as the historical development of these models. They conclude that the mode age *M* increases over time. We follow these results and observations as well as the statements from Gavrilov and Gavrilova [7] and incorporate mortality improvement into Model (1) via

(2) $$\mu_{\vartheta }\left(x,t\right)=\left(\alpha +\beta \;e^{\beta (x-M)}\right)e^{-\lambda (t-2020)},$$

where mortality decreases with a rate λ per calendar year. In (2), *t* is the calendar year (t=2023, 2022, etc.), and the reference year 2020 (more precisely, the reference date 2020, Jan. 1st) is chosen for convenience. It does not impact the mortality results. Note that (2) is (with $\alpha_{t}=\alpha \;\cdot \;e^{-\lambda (t-2020)}$)

(3) $$\mu_{\vartheta }\left(x,t\right)=\alpha_{t}+\beta \;e^{\beta (x-M_{t})},$$

where $M_{t}:=M+\frac{\lambda }{\beta }\left(t-2020\right)$ is the life expectancy, growing with λ/β year by year. Proposition A1 in the Appendix B relates the parameters of Model (2) with further characteristics of the population as the mode.

### 2.3. Estimation of Parameters

The parameters describing the force of mortality in Model (2) are ϑ=(α,β,M,λ). The least-squares approach involves $E_{x}$, that is, the number of individuals at age *x*, out of which $D_{x}$ die within the observation period. Model (2) with parameters ϑ=(α,β,M,λ) estimates the probability of an individual at age *x* to die in the following year by $q_{\vartheta ;x}$, which relates to the force of mortality by *q_x_* (cf. (A1) in Appendix A and Richards [23]). The least-squares approach compares the observed deaths ($D_{x}$) with expected deaths ($q_{x}\;\cdot \;E_{x}$) and considers

(4) $$\frac{(D_{x}-q_{x}\;\cdot \;E_{x}{)}^{2}}{q_{x}\;\cdot \;E_{x}}\;\;\mathrm{or}\;\;\frac{(E_{x}-D_{x}-\left(1-q_{x}\right)\;\cdot \;E_{x}{)}^{2}}{\left(1-q_{x}\right)\;\cdot \;E_{x}},$$

where the symmetric, second term in (4) relates the observed survivals $E_{x}-D_{x}$ with the expected survivals $\left(1-q_{x}\right)\;\cdot \;E_{x}$. The least-squares objective to identify the optimal parameters ϑ combines both and is

(5) $$\sum_{x\geq 0}\frac{{\left(D_{x}-q_{x}\;\cdot \;E_{x}\right)}^{2}}{q_{x}\;\cdot \;E_{x}}+\frac{(E_{x}-D_{x}-\left(1-q_{x}\right)\;\cdot \;E_{x}{)}^{2}}{\left(1-q_{x}\right)\;\cdot \;E_{x}}=\sum_{x\geq 0}{\left(\frac{D_{x}-q_{x}\;\cdot \;E_{x}}{\sqrt{q_{x}\left(1-q_{x}\right)\;\cdot \;E_{x}}}\right)}^{2},$$

where the sum is among all ages considered in estimating the parameters. This estimation technique to identify the parameters is consistent with the following analysis and complies with the $\chi^{2}$ test and the G test, respectively.

### 2.4. Analysis of Excess Mortality

To investigate excess mortality in year *t* for specific ages, we consider the quantity termed *Z-score*, which is genuinely present in (5). In contrast to the popular definition of the *Z*-score, we define the score by involving the estimated mean and study

(6) $$Z_{x}^{t}:=\frac{D_{x}^{t}-q_{x}^{t}\;\cdot \;E_{x}^{t}}{\sqrt{\left(1-q_{x}^{t}\right)q_{x}^{t}\;\cdot \;E_{x}^{t}}}$$

(cf. (Equation (6.1)) in Cairns et al. [26] for a similar quantity) for individual ages *x* and calendar years *t*.

The *Z*-score has the following properties, which are crucial for the analysis of excess mortality:

(i) $Z_{x}^{t}>0$ if more deaths are recorded than expected, and $Z_{x}^{t}\gg 1$ (i.e., $Z_{x}^{t}$ much greater than 1), which indicates excess mortality for the group with age *x*;
(ii) The score in (6) is sensitive with respect to the estimated mean, and even vanishes for the crude estimator ${\hat{q}}_{x}^{t}=\frac{D_{x}^{t}}{E_{x}^{t}}$;
(iii) As follows from the central limit theorem, the quantity (6) is asymptotically normally distributed with standard parameters (Z∼N(0,1)) for the true parameter $q_{x}^{t}$;
(iv) For the estimate ${\hat{q}}_{\vartheta ;x}$, the score (6) is the crucial quantity in our following analysis, as positive aberrations (i.e., $D_{t}^{x}\gg q_{x}^{t}\;E_{x}^{t}$) indicate excess mortality for the specific age *x*;
(v) The squared *Z*-score is the building block of the $\chi^{2}$ test, as building block of the $\chi^{2}$ test, as
  $$T_{x}^{t}:=\frac{{\left(D_{x}^{t}-E_{x}^{t}{\hat{q}}_{\vartheta ;x}^{t}\right)}^{2}}{E_{x}^{t}\;{\hat{q}}_{\vartheta ;x}^{t}}+\frac{{\left(E_{x}^{t}-D_{x}^{t}-E_{x}^{t}\left(1-{\hat{q}}_{\vartheta ;x}^{t}\right)\right)}^{2}}{E_{x}^{t}\;\left(1-{\hat{q}}_{\vartheta ;x}^{t}\right)}={\left(Z_{x}^{t}\right)}^{2},$$
  cf. (5) above. It follows that the test quantity
  (7) $$T^{t}:=\sum_{x=0}T_{x}^{t}=\sum_{x=0}{\left(Z_{x}^{t}\right)}^{2}$$
  follows a $\chi^{2}$ distribution, where the degree of freedom is the number of groups of age the sum in (7) takes into account.

## 3. Results

### 3.1. Estimators and Model Validation

#### 3.1.1. Parameters Characterizing the Mortality of the German Population

The empirical mortality rates depicted in Figure 1 for different calendar years support Model (2). Table 1 presents the parameters of the model found for the German population. The parameters indicate that women live longer than men, as their mode age *M* exceeds men’s mode. In contrast, the baseline probability α (the Makeham term) is somewhat lower for men than for women, but this quantity only has a minor influence on the overall mortality. Mortality gradually improves over time with rate λ=1.73% for women and λ=2.26% for men, i.e., the survival probabilities improve faster for men, while survival probabilities for women are more saturated. From the parameters in Table 1, it is evident that the mortality rates of men and women differ. Our analysis gives plausible results, and we investigate the sexes separately.

#### 3.1.2. Mortality Trend

In a long-term study including many countries of the Western world, Rischatsch et al. [28] (p. 2) report that mortality improves by approximately 1–2% per year for both sexes. This trend is in line with our results for λ in Table 1, where the mortality of men in our sample improves slightly faster. Moreover, we want to point out that, following Figure 1 in Rischatsch et al. [28], the average life expectancy increased from roughly 40 years in 1860 to about 80 years in 2016 for countries of the Western world, which is an improvement of approximately 26%, i.e., 26 years of improved life expectancy per century for both sexes. Following (A2) in Proposition A1 (Appendix B), the mode improves at the rate $\frac{\lambda }{\beta }$, for which Table 1 reveals the quantities

$$\begin{array}{cc} \; & \lambda /\beta \approx 14\;\mathrm{years}\;\mathrm{per}\;\mathrm{century}\;\mathrm{for}\;\mathrm{women},\;\mathrm{and}\;\\ \; & \lambda /\beta \approx 21\;\mathrm{years}\;\mathrm{per}\;\mathrm{century}\;\mathrm{for}\;\mathrm{men} \end{array}$$

for Germany. These external sources thus confirm our results and conclusion that mortality improvement rates must not be neglected.

**Figure 2:**
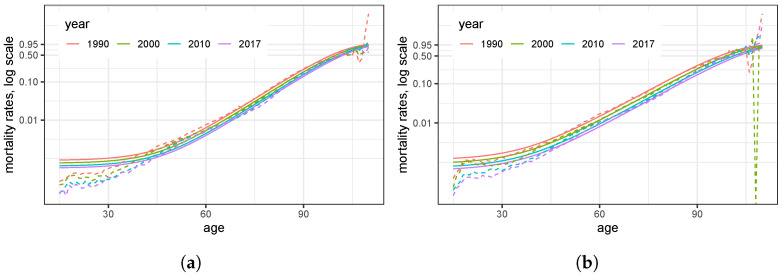
One-year probabilities ${\hat{q}}_{x}$ and the fitted model ${\hat{q}}_{\vartheta ;x}$, derived from the data source, displayed on logarithmic scale: (**a**) women; (**b**) men.

Figure 2 displays derived 1-year mortalities ${\hat{q}}_{\vartheta ;x}$ based on the parametric model (2) for selected observation years together with the rough estimates ${\hat{q}}_{x}$ from Figure 1. The deviations for persons dying before reaching 30 years are small and in the range of a few per million, while the deviations for very high ages are caused by the very few persons dying at these high ages. These figures, along with the literature referenced above, provide evidence for the modeling approach chosen, i.e., for Model (2).

**Figure 3:**
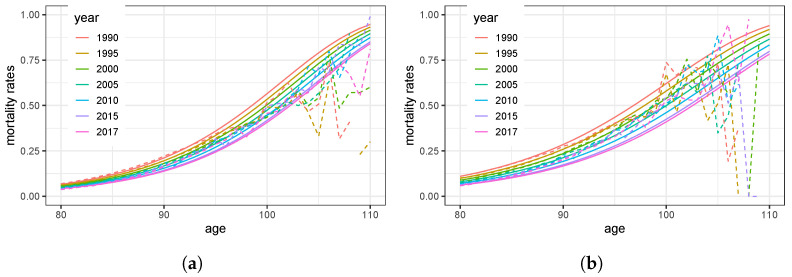
Empirical and estimated mortality rates of elderly people: (**a**) women; (**b**) men.

The illustrations in Figure 3 highlight older ages. The rough estimator ${\hat{q}}_{x}=\frac{D_{x}}{E_{x}}$ and the smoothed version ${\hat{q}}_{\vartheta ;x}$ coincide up to the age of 90 for women and even up to the age of 100 for men. For extremely high ages, the rough estimators $\hat{q}x$ fluctuate more due to narrowing data. The chart in Figure 3b for men displays a larger bandwidth than Figure 3a, which is a consequence of the rate λ driving the trend of decreasing mortality.

**Figure 4:**
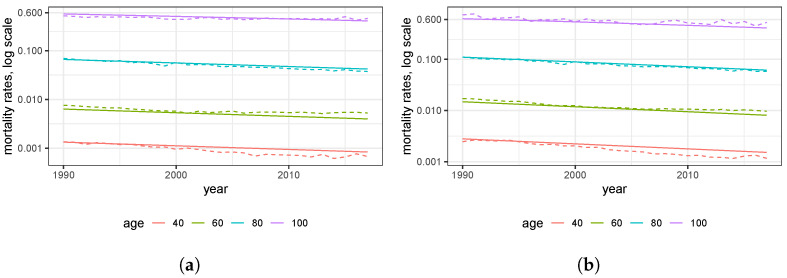
Empirical and estimated trend of mortality rates over the time period 1990 to 2019 for selected ages: (**a**) women; (**b**) men.

Figure 4 highlights the mortality trend for specific ages (cf. Proposition A1). These rates decrease mortality linearly over time on the logarithmic scale. Further, the decrease observed is overall uniform among all ages displayed. Again, this provides evidence in favor of the parameter λ in Model (2).

### 3.2. *Z*-Scores of the Training Data

**Figure 5:**
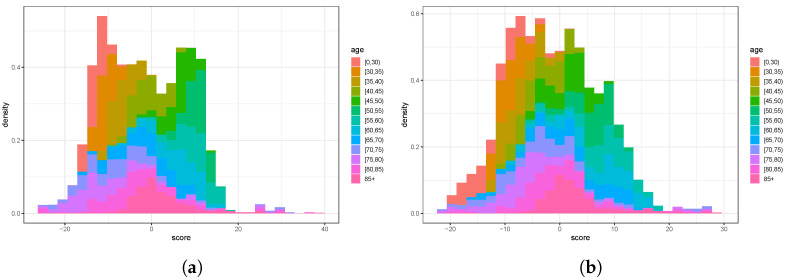
Histograms of age-specific *Z*-scores of the training data, 1990–2017: (**a**) female; (**b**) male.

The histogram of age-specific *Z*-scores (6) for the parameter ${\hat{q}}_{x}^{t}$ (Figure 5) exhibits unobserved heterogeneity (i.e., overdispersion), specifically a standard deviation of approximately 12.4 for women and about 12.2 for men. Given that, the score in (6) can be rearranged to

$${\hat{q}}_{x}-\frac{D_{x}^{t}}{E_{x}^{t}}\;\approx \;\pm 12\;{\left(\frac{(1-{\hat{q}}_{x}){\hat{q}}_{x}}{E_{x}^{t}}\right)}^{1/2}.$$

This reveals a quantitative relation of the empirical estimator ${\hat{q}}_{x}^{t}=D_{x}^{t}/E_{x}^{t}$ and the estimator ${\hat{q}}_{\vartheta ;x}^{x}$ of the parametric model. Due to the term $\sqrt{\hat{q}\left(1-\hat{q}\right)}$, it is apparent as well that the estimators tend to deviate less with increasing $E_{x}^{t}$ and with the mortality parameter close to the boundary, i.e., with young and very old people.

The following section relates the computational results to the COVID-19 years 2020 to 2022.

## 4. Discussion

### 4.1. Historic Mortality Rates, Compared to COVID-19 (2020 to 2023)

Figure 5 visualizes *Z*-scores for different age groups. The plots expose different locations and widths for varying age groups. Recall that aberrations to the right indicate excess mortality (cf. (6) and Points (i) and (iv) on page 4).

**Figure 6:**
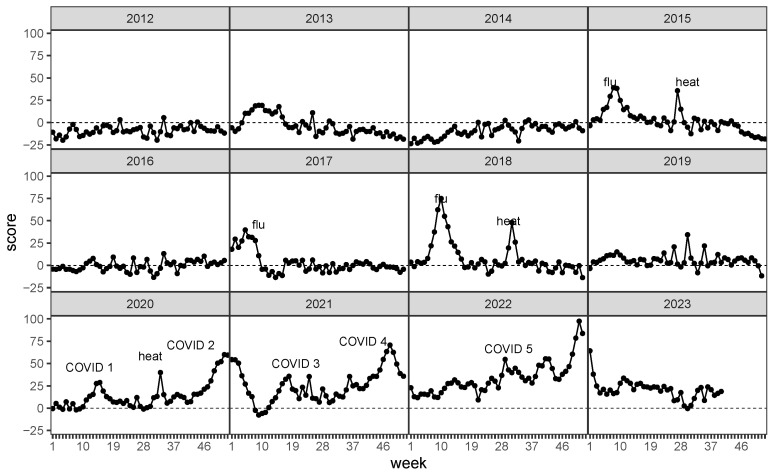
Excess mortality index of the entire population, 2000–2023. Highlighted are COVID-19, flu, and heat waves.

Figure 6 displays the evolution of the score over time. The figure exhibits excess mortality due to

- COVID-19;
- heat waves in summer;
- flu waves in winter.

Over the years, the scores have fluctuated around 0, but the COVID-19 years have notably changed this behavior. Except for one month in 2021, Figure 6 displays a strictly positive *Z*-score in more than two years. Once again, it is a strict indicator for excess mortality in the past years from 2020 to 2022. Analysis of the current year data 2023, which is still incomplete, shows that excess mortality has regressed. The minor deviations of the score from the zero line are negligible. In addition, however, the proposed score also shows excess mortality during influenza waves in 2015, 2017 and in particular throughout the severe flu season in 2018, as well as during periods of severe heat in the summer.

### 4.2. Mortality of the Elderly Population

**Figure 7:**
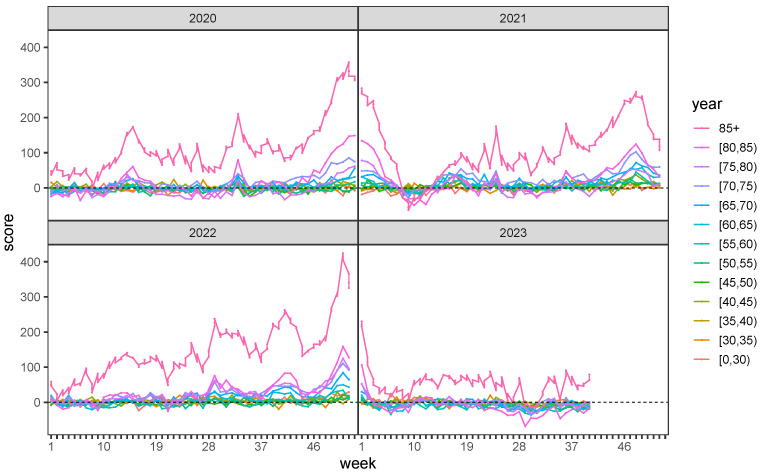
Weekly scores $Z_{jx}^{t}$ (2020–2023, cf. (6)) for individual age groups.

Figure 7 details excess mortality for different age groups. It is evident from the picture that it is the elderly segment of the German population that is primarily impacted by excess mortality. More specifically, excess mortality starts to affect the segment of people aged 60 or older.

The first peak, being the highest at the end of winter 2018, originates from a heavy flu epidemic, which caused excess mortality of 27,000 deaths within a week, compared to 20,000 deaths in the same week in prior years. The second peak at the end of 2020 is caused by COVID-19; this trend continues in the first months of 2021. During this period, the mortality exceeds 24,000 deaths per week, which is unprecedented in the previous decades. It should be noted that this excess mortality was documented despite the fact that the country was in partial or full lockdown over several weeks. It is evident that the total mortality, without governmental countermeasures such as the lockdown, would have been higher. Keeping in mind that fatal accidents related to traffic, workplace or leisure were much lower during that time, yet, the mortality was higher than in comparable years without COVID-19. For the integrity of the argument we emphasize that our analysis only assesses total mortality, i.e., we do not take the medical cause of death into consideration. In fact, we do not distinguish between individuals who were diagnosed with COVID-19 or died of COVID-19 or of other causes. All conclusions are based on total mortality.

## 5. Conclusions

We investigated excess mortality during the COVID-19 pandemic in 2020 to 2022 in Germany. For the analysis, we examined a specific indicator, the *Z*-score, which is designed to uncover excess mortality. Based on the limited data available during the pandemic we recorded, detected, and measured excess mortality, specifically for the elderly segment of the population.

## Figures and Tables

**Table 1:** Parameters describing the mortality of the German population.

	Mode *M* in Years	α	β	λ/ Year	λ/β%
women	89.57	5.29	12.56%	1.73%	13.74
men	86.09	5.86	10.64%	2.26%	21.23

Explanations of parameters: *M* is the base parameter for population’s mode age of death. α is the base mortality rate, independent on age *x*. β is the rate of mortality increase by age, cf. (Chapter 9 in [27]) or [5]. λ is the mortality trend improving over time. λ/β is the population’s mortality improvement in years per century.

## Data Availability

The model was fitted with data from 1990 to 2017 provided by the Human Mortality Database [30]. The excess mortality index uses the population figures and life tables [21,22] published annually by the Federal Statistical Office to calculate expected deaths and compares them with the actual deaths [20], which are also published by the Federal Statistical Office.

## Appendix A

The force of mortality (also known as hazard function in reliability theory) corresponding to an $R_{\geq 0}$-valued random variable with cumulative, differentiable distribution function *F* with density $f=F^{\prime }$ (the derivative) is

(A1) $$\mu \left(x\right):=\frac{F^{\prime }\left(x\right)}{1-F(x)}=-\frac{d}{dx}\ln\left(1-F(x)\right),$$

cf. Richards [23].

## Appendix B

**Proposition A1** (Mode)**.** *The mode of the distribution with time force of mortality (2) is*

(A2) $$M-\frac{2\alpha }{\beta^{2}}+\frac{\lambda }{\beta }\left(t-2020\right)+\varepsilon \left(\alpha \right),$$

*where the error $\left|\varepsilon \left(\alpha \right)\right|\leq C\;\cdot \;\alpha^{2}$ for a real constant C (and hence negligible for small values of α, as is the case in our application, cf. Table 1).*

**Proof of Proposition** **A1.** The density attains its maximum at $x_{m}$ if $F^{\prime \prime }\left(x_{m}\right)=0$ and the characterizing equation

$$\mu^{\prime }\left(x_{m}\right)=\mu {\left(x_{m}\right)}^{2}$$

follows from

(A3) $$F^{\prime }\left(x\right)=\mu \left(x\right)\;e^{M(0)-M(x)}$$

with $M\left(x\right):=\int_{x_{0}}^{x}\mu \left(s\right)\;ds$. Based on the force of mortality (2), define the function

(A4) $$\begin{array}{cc} g(x;\alpha ) & :=\mu_{\alpha ,\beta ,M,\lambda }{\left(x\right)}^{2}-\mu_{\alpha ,\beta ,M,\lambda }^{\prime }\left(x\right)\\ & ={\left(\alpha +\beta \;e^{\beta (x-M)}\right)}^{2}\;e^{-2\lambda (t-2020)}-\beta^{2}\;e^{\beta (x-M)-\lambda (t-2020)} \end{array}$$

and observe that

$$g\left(M+\frac{\lambda }{\beta }\left(t-2020\right);0\right)=0.$$

It follows that $x_{m}=M+\frac{\lambda }{\beta }\left(t-2020\right)$ is the mode. The derivative of (A4) with respect to *x* is

$$\begin{array}{cc} \frac{\partial }{\partial x}g\left(x;\alpha \right) & =2\left(\alpha +\beta \;e^{\beta (x-M)}\right)e^{-2\lambda (t-2020)}\;\beta^{2}e^{\beta (x-M)}-\beta^{3}\;e^{\beta (x-M)-\lambda (t-2020)}, \end{array}$$

and with respect to α

$$\begin{array}{cc} \frac{\partial }{\partial \alpha }g\left(x;\alpha \right) & =2\left(\alpha +\beta \;e^{\beta (x-M)}\right)e^{-2\lambda (t-2020)}. \end{array}$$

Evaluated at $x=M+\frac{\lambda }{\beta }\left(t-2020\right)$ and α=0, these quantities are

$$\frac{\partial }{\partial x}g\left(M+\frac{\lambda }{\beta }\left(t-2020\right);0\right)=2\beta^{3}-\beta^{3}$$

and

$$\frac{\partial }{\partial \alpha }g\left(M+\frac{\lambda }{\beta }\left(t-2020\right);0\right)=2\beta .$$

By the implicit function theorem, it follows that the solution $x_{m}\left(\alpha \right)$ of $g\left(x_{m}\left(\alpha \right);\alpha \right)=0$ has derivative

$$\frac{d}{d\alpha }x_{m}\left(\alpha \right)=-\frac{\frac{\partial }{\partial \alpha }g}{\frac{\partial }{\partial x}g}=-\frac{2}{\beta^{2}}$$

and hence the assertion (A2). □
